# Clustering of the causes of death in Northeast Iran: a mixed growth modeling

**DOI:** 10.1186/s12889-023-16245-y

**Published:** 2023-07-19

**Authors:** Nasrin Talkhi, Zohreh Emamverdi, Jamshid Jamali, Maryam Salari

**Affiliations:** Department of Biostatistics, School of Health, Social Determinants of Health Research Center, Mashhad University of Medical Sciences, Mashhad, Iran

**Keywords:** Cause of death, Mortality, Cluster analysis, International classification of diseases, Latent growth mixture models, Iran

## Abstract

**Background:**

Processing and analyzing data related to the causes of mortality can help to clarify and monitor the health status, determine priorities, needs, deficiencies, and developments in the health sector in research and implementation areas. In some cases, the statistical population consists of invisible sub-communities, each with a pattern of different trends over time. In such cases, Latent Growth Mixture Models (LGMM) can be used. This article clusters the causes of individual deaths between 2015 and 2019 in Northeast Iran based on LGMM.

**Method:**

This ecological longitudinal study examined all five-year mortality in Northeast Iran from 2015 to 2019. Causes of mortality were extracted from the national death registration system based on the ICD-10 classification. Individuals' causes of death were categorized based on LGMM, and similar patterns were placed in one category.

**Results:**

Out of the total 146,100 deaths, ischemic heart disease (21,328), malignant neoplasms (17,613), cerebrovascular diseases (11,924), and hypertension (10,671) were the four leading causes of death. According to statistical indicators, the model with three classes was the best-fit model, which also had an appropriate interpretation. In the first class, which was also the largest class, the pattern of changes in mortality due to diseases was constant (*n* = 98, 87.50%). Second-class diseases had a slightly upward trend (*n* = 10, 8.92%), and third-class diseases had a completely upward trend (*n* = 4, 3.57%).

**Conclusions:**

Identifying the rising trends of diseases leading to death using LGMM can be a suitable tool for the prevention and management of diseases by managers and health policy. Some chronic diseases are increasing up to 2019, which can serve as a warning for health policymakers in society.

**Supplementary Information:**

The online version contains supplementary material available at 10.1186/s12889-023-16245-y.

## Introduction

Death is an inevitable and natural occurrence that can be attributed to a multitude of factors, including but not limited to cancers, chronic diseases, HIV, communicable and non-communicable diseases, accidents, drug abuse, suicide, traumatic injuries, complications during childbirth, stroke, Alzheimer's disease, and unhealthy lifestyle choices. The identification of commonalities among diseases has facilitated their classification [1]. The World Health Organization (WHO) has established the International Classification of Diseases (ICD) as a comprehensive medical classification system that encompasses codes for diseases, signs and symptoms, abnormal findings, complaints, social circumstances, and external causes of injury or diseases [1, 2]. Due to the high prevalence and significant variability of mortality rates across different geographical regions, identifying the precise causes of death can be challenging and, in some cases, impossible [3].

A published study on the Iranian population aimed at identifying the most prevalent causes of death. The results revealed that Cardiovascular Disease (CVD) was the leading cause of death, followed by motor vehicle accidents. Additionally, cancers and intentional and unintentional injuries were also identified as common causes of death [4]. Overall, there has been a significant increase in mortality rates in Iran [5]. The various provinces of Iran, such as Razavi-Khorasan, exhibit different financial, geographical, cultural, and social conditions, as well as lifestyles. These factors have been identified as significant determinants in the incidence of diseases and, in turn, mortality rates [6]. It is important to note that the causes of death can be influenced by genetic characteristics, lifestyle choices, living environment, and demographic factors. Furthermore, these causes can vary significantly across cities and countries [7].

The health department of the Ministry of Health in Iran maintains the most comprehensive system for registering causes of death. This system draws information from various sources, such as mortuaries, forensic medicine, academic, and non-academic hospitals. The data used in this study were extracted from this system.

Analyzing data and exploring important patterns or trajectories in various causes of death can help to decrease the mortality rate and increase the life expectancy in a society [8].

Prevalence or descriptive studies are considered less valuable compared to analytical-descriptive studies as they solely offer a description of the current state of affairs [9]. Traditional approaches, such as repeated measures analysis of variance and multivariate analysis of variance, possess limitations when it comes to analyzing longitudinal data [10–12]. The latent growth model is one of the methods developed for analyzing longitudinal data, aiming to overcome the limitations of traditional approaches. By employing latent factors, this model can effectively determine the pattern of the response variable [13]. The significance of the present study lies in the utilization of an advanced analytical-descriptive method, specifically the linear/non-linear growth mixture model (GMM) technique. This approach allows for the investigation of disease trends within various subgroups, while simultaneously evaluating the overall disease trend. GMM serves as a latent categorical variable model that identifies unobserved heterogeneity within a population, enabling the identification of groups of individuals who share similar growth trajectories [14]. Therefore, the objective of this study was to employ linear GMM (LGMM) to cluster the causes of death among individuals in Northeast Iran between 2015 and 2019, based on the 10th Revision of ICD (ICD-10). Additionally, supplementary analyses were conducted to examine the data within gender and age groups.

## Material and methods

### Data description

The data utilized in this ecological longitudinal study were collected between 2015 and 2018. Mortality was measured using death certificates, which serve as medical certificates detailing the causes of death [9, 15]. In this study, the causes of death, including underlying diseases, were extracted from death certificates registered in the electronic death registration system of Razavi-Khorasan province in Iran. The study population consisted of all individuals living in Razavi Khorasan whose cause of death was recorded in the system of the Vice-Chancellor of Health at Mashhad University of Medical Sciences. Additionally, the population of Razavi Khorasan was approximately 7,400,000 individuals [16]. It is the most comprehensive system in Iran for registering the causes of death. This system operates under the health department of the Ministry of Health and receives information from various sources, including mortuaries, forensic medicine, academic, and non-academic hospitals. The data from various sources, including mortuaries, forensic medicine, academic, and non-academic hospitals, were merged. During the data cleaning and preprocessing phase, records without recorded or missing cause of death/ICD-10 codes, as well as illegible, irrelevant, and duplicated data, were excluded. The process of editing, correcting, coding, label encoding, and structuring the data within the dataset was carefully reviewed and performed. Following these actions, the data with acceptable values were prepared for the data analysis phase. Following the completion of the data cleaning phase, the cause of death data for a total of 142,896 individuals was available. As our objective was to classify the causes of death, we utilized the frequencies of each cause observed over a span of five years. Causes of death with a frequency below 30 were disregarded in the analysis. Consequently, our investigation encompassed the analysis of 112 distinct categories of causes of death during this five-year period.

### Data analysis

Understanding patterns, growth, ascents, or declines in behavior or specific processes is frequently a subject of interest for psychologists and social scientists. Among the analytical tools available, growth curve models prove to be valuable and practical for capturing systematic changes over time. This model enables the study of both intra and inter-individual changes in longitudinal data spanning several years [17].

In real-time and applied research aimed at exploring intra and inter-individual change, growth mixture models are commonly employed [18, 19]. The LGMM model takes into account the variability of patterns of change over time both between subjects and within subjects [19]. Linear and non-linear trajectories can be identified using growth curve models as well as LGMM. In other words, the LGMM model probabilistically assigns subjects or patients into latent classes over time or using longitudinal data, with the aim being to achieve this classification [20]. In the presence of unobserved heterogeneity in populations, the use of Growth Mixture Modeling (GMM) is a practical approach for identifying such heterogeneity [18].

**Fig. 1:**
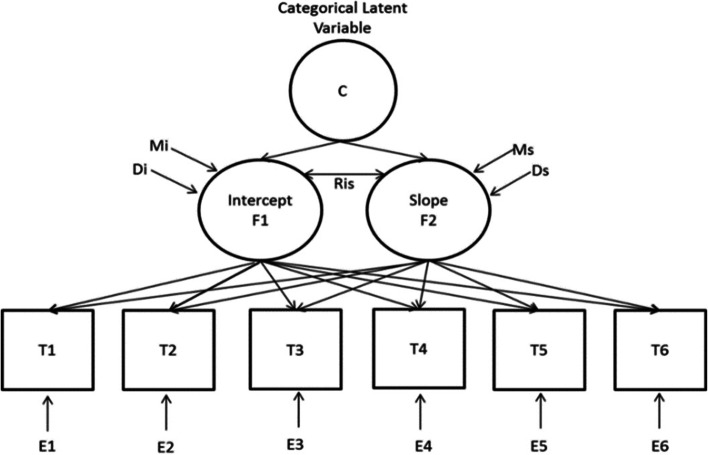
General diagram of the growth mixture model used in the study

The objective of the Latent Growth Model (LGM) is to investigate longitudinal changes in a sample of data. The GMM is an extension of the LGM that describes longitudinal changes in heterogeneous subgroups or subsamples within a dataset [21]. In the LGMM theory, one intercept and slope (considered as a latent variable) is estimated for each subject, which is allowed to vary across subjects. The estimated variance of the intercept and slope variables indicates variability across subjects [22]. Additionally, the modeled intercept and slope in the GMM model are represented as baseline values and rates of change. In other words, it shows how the subject's baseline values relate to their rate of change [22]. The intercept and slope of all subjects can be summarized as an average with means of latent variables. In other words, the means of latent variables reflect the average of all subject's intercepts and slopes. As mentioned above, the LGMM model considers the desired number of time points. At each time point, it is possible that subjects deviate from their mean, which is referred to as error or residual variances [22]. The LGMM is graphically depicted in Fig. 1. To specify the optimal latent classes, various indices such as relative fit information criteria, including the Akaike Information Criterion (AIC), Bayesian Information Criterion (BIC), Entropy (a statistic that ranges from zero to one), and Likelihood Ratio Test (LRT) were used. Lower values of AIC and BIC suggest a better model fit. A high value of entropy indicates that subjects are adequately separated with confidence, and a significant P-value in the LRT test indicates a model with k classes fits the data better.

## Results

### Statistical analysis

**Fig. 2:**
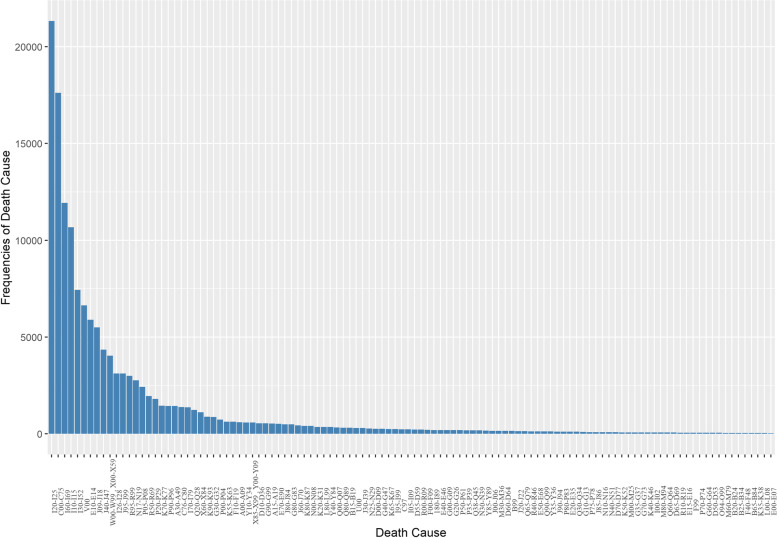
The frequencies of death cause in the northeast of Iran in 2019

**Fig. 3:**
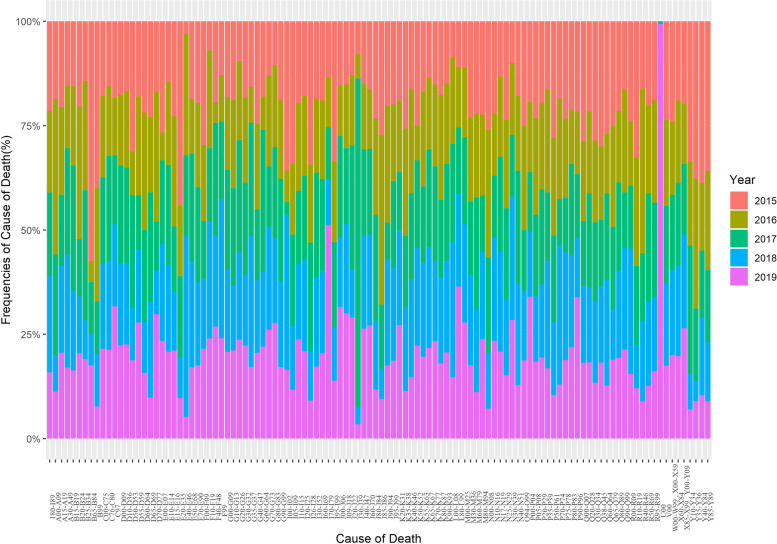
Bar chart for death causes’ frequencies (%) in northeast of Iran during 2015–2019

**Fig. 4:**
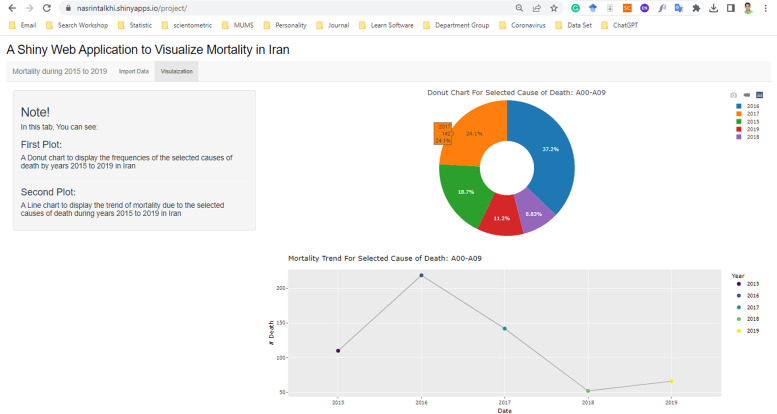
A view of the interactive web application with shiny to visualize and describe data

Statistical analyses were conducted using R statistical software version 4.1.1. Figures 2 and 3 display the frequencies of deaths that occurred between 2015 and 2019. Additional descriptions of the data can be found at the https://nasrintalkhi.shinyapps.io/project/ URL link, and Fig. 4 illustrates a view of this web application.

In 2019, the four leading causes of mortality in the northeast of Iran were ischemic heart diseases (I20-I25) with 21,328 cases, determined and presumed primary malignant neoplasms in special locations except for lymph, hematopoietic system, and related tissue (C00-C75) with 17,613 cases, vascular diseases of the brain (I60-I69) with 11,924 cases, and high blood pressure disease (I10-I15) with 10,671 cases. These causes had a significantly higher frequency compared to other causes of death. Forms of heart diseases (I30-I52), traffic accidents and transportation (V00), diabetes mellitus (E10-E14), and influenza and pneumonia (J09-J18) were the next four important causes of death in 2019, with 7,441, 6,631, 5,888, and 5,495 cases, respectively. Other causes of death were less frequent.

Forms of heart diseases (I30-I52), traffic accidents and transportation (V00), diabetes mellitus (E10-E14), and influenza and pneumonia (J09-J18) with 7,441, 6,631, 5,888, and 5,495 cases were the next four important death causes in 2019, respectively. Other causes are in the next levels. Special cases (U00) had a considerable frequency (293 cases) compared to four years ago (2015–2018). Other diseases of the upper respiratory tract (J30-J39) had a significant frequency (230 cases) in 2017 so before and after 2017 experienced a sharp increasing and decreasing pattern, respectively. Diseases of arteries, arterioles, and capillaries (I70-I79) had a constant trend from 2015 to 2018, while they had a sharp increasing trend in 2019. Diseases caused by intestinal worm parasites (B65-B84) with 23 cases experienced most its value in 2015 and then it had an almost decreasing pattern. Frequencies of other causes were reported in more detail in Table A1.

### Linear growth mixture model

In this study, existing trajectories were identified using Latent Growth Mixture Modeling (LGMM) from 2015 to 2019. To establish a linear growth model, equidistant time scores were set as 0, 1, 2, 3, and 4. The non-linear LGMM was examined, but the quadratic term did not yield the best fit. Consequently, the linear LGMM was employed and extended to include models with 1 to 3 classes. Model comparison indices, including AIC, BIC, entropy, and *p*-values from the likelihood ratio test, were reported in Table 1 to determine the model that best fit the data.

**Table 1:** Fitted indices for unconditional LGMMs with 1 to 3 classes

Class	AIC	BIC	Entropy	*P*-value (LRT test)	Percent in class		
					**Class1**	**Class2**	**Class3**
**1**	8821.01	8833.88	1.00	-	100%		
**2**	8115.38	8136.41	1.00	< 0.001*	3.57%	96.42%	
**3**	7747.67	7709.29	0.996	< 0.001*	87.50%	8.92%	3.57%

* significant in 0.05 error level

As observed, the AIC, BIC, and entropy values decrease as the number of classes increases. For clustering with 1 linear class, the AIC was 8821.01, BIC was 8833.88, and entropy was 1. Clustering with 2 linear classes resulted in an AIC of 8115.38, BIC of 8136.41, and entropy of 1. Finally, for clustering with 3 linear classes, the AIC was 7747.67, BIC was 7709.29, and entropy was 0.996. The likelihood ratio test (LRT), with the null hypothesis (H0) of k classes and the alternative hypothesis (H1) of k + 1 classes, was significant in all cases. Previous studies have indicated that when there is a discrepancy between the LRT test and relative fit information criteria, the BIC criterion should be given greater consideration. Additionally, interpretability is an important factor in selecting the optimal number of classes. Considering the goodness of fit indices for models with 1–3 classes, the percentage of membership to each class, and interpretability, the model with 3 classes was chosen as the optimal choice. The majority of death causes were allocated to the 1st class (Table 2).

**Table 2:** Allocated death causes into classes 1 to 3 using LGMM

Class	ICD-10 code	Disease full name	ICD-10 code	Disease full name	ICD-10 code	Disease full name
**Class 1**	A00-A09	Intestinal infectious diseases	G40-G47	Occasional and epizootic diseases	N00-N08	Glomerular diseases
	A15-A19	Tuberculosis	G60-G64	Polyneuropathies and peripheral nerve damage	N10-N16	Tubulointerstitial diseases
	A30-A49	Other bacterial diseases	G70-G73	Diseases of neuromuscular junction	N25-N29	Other kidney and ureter disorders
	B15-B19	Viral hepatitis	G80-G83	Cerebral palsy and other paralytic syndromes	N30-N39	Other urinary system disorders
	B20-B24	HIV acquired immunodeficiency patients	G90-G99	Other nervous system disorders	N40-N51	Disorders of male sexual organs
	B25-B34	Other viral diseases	I00-I02	Acute rheumatic fever	O94-O99	Other pregnancy conditions not classified elsewhere
	B65-B84	Diseases caused by intestinal worm parasites	I05-I09	Chronic rheumatic heart disease	P00-P04	Maternal factors affecting the fetus and newborn caused by complications of pregnancy and childbirth
	B99	Other infectious diseases	I70-I79	Diseases of arteries, arterioles and capillaries	P05-P08	Fetus and newborn problems caused by the length of the pregnancy period or the growth of the fetus in the mother's womb
	C76-C80	Malignant neoplasms of other secondary and unspecified locations	I80-I89	Diseases of veins, lymphatic vessels and lymph nodes not classified elsewhere	P20-P29	Cardiovascular and respiratory disorders of the fetus or newborn that are related to the period around birth
	C97	Independent malignant neoplasms (primary) of multiple locations	I95-I99	Other disorders and unspecified disorders of circulatory system	P35-P39	Infectious disorders of the newborn or fetus that are related to the period around birth
	D00-D09	Neoplasm in situ	J00-J06	Acute upper respiratory infections	P50-P61	Blood and coagulation disorders of the newborn or fetus that are related to the period around birth
	D10-D36	Benign neoplasm	J20-J22	Other acute lower respiratory infections	P70-P74	Transient endocrine and metabolic disorders of the fetus or newborn that are related to the period around birth
	D50-D53	Nutritional anemias	J30-J39	Other diseases of the upper respiratory tract	P75-P78	Gastrointestinal disorders of the fetus or newborn that are related to the period around birth
	D55-D59	Hemolytic anemias	J60-J70	Pulmonary diseases caused by external factors	P80-P83	Skin conditions and temperature regulation of the fetus or newborn that is related to the period around birth
	D60-D64	Aplastic anemia and other anemias	J80-J84	Other respiratory diseases affecting interstitial tissue	P90-P96	Other causes of death around birth
	D65-D69	Coagulation defects, purpura and other bleeding conditions	J85-J86	Infectious and necrotic conditions of the lower respiratory system	Q00-Q07	Congenital defects of the nervous system
	D70-D77	Other diseases of blood and hematopoietic organs	J90-J94	Other pleural membrane diseases	Q20-Q28	Congenital defects of the circulatory system
	E00-E07	Thyroid gland disorders	K20-K31	Diseases of esophagus, stomach and duodenum	Q30-Q34	Congenital defects of the respiratory system
	E15-E16	Regulation and pancreatic internal secretion	K35-K38	Diseases of the appendix	Q38-Q45	Other congenital defects of digestive system
	E20-E35	Disorders of other endocrine glands	K40-K46	Herni	Q60-Q64	Congenital defects of the urinary system
	E40-E46	Malnutrition	K50-K52	Enteritis and non-infectious colitis	Q65-Q79	Congenital defects and deformities of the musculoskeletal system
	E50-E68	Other nutritional deficiencies of obesity and other overeating	K55-K63	Other intestinal diseases	Q80-Q89	Other congenital defects
	E70-E90	Metabolic disorders	K65-K67	Peritoneal diseases	Q90-Q99	Chromosomal disorders not classified elsewhere
	F00-F09	Organic disorders including mental symptoms	K70-K77	Liver diseases	R00-R09	Signs and symptoms related to the cardiorespiratory system
	F10-F19	Mental behavioral disorders caused by the consumption of psychotropic drugs	K80-K87	Disorders of the gallbladder, bile ducts and pancreas	R10-R19	Signs and symptoms related to digestive system
	F40-F48	Neurotic, stress-related and somatiform disorders	K90-K93	Other gastrointestinal diseases	R40-R46	Signs and symptoms of cognitive, understanding, emotional and behavioral states
	F99	Unspecified mental disorder	L00-L08	Skin and subcutaneous tissue infection	R50-R69	General signs and symptoms
	G00-G09	Inflammatory diseases of the central nervous system	L80-L99	Other skin and subcutaneous tissue disorders	U00	Special cases
	G10-G13	Systemic atrophy that primarily involves the central nervous system	M00-M25	Arthropathies	X60-X84	Suicide (self-harm)
	G20-G26	Extrapyramidal movement disorders	M30-M36	Systemic connective tissue disorders	X85-X99, Y00-Y09	Murder
	G30-G32	Other degenerative diseases of nervous system	M60-M79	Soft tissue disorders	Y10-Y34	Other abuse
	G35-G37	Demilitarization diseases of the central nervous system	M80-M94	Joint and bone disorders	Y35-Y36	Legal intervention and war operations
	Y40-Y84	Complications from medical and surgical care	Y85-Y89	Consequences, complications, external causes of disability and mortality		
**Class 2**	E10-E14	Diabetes mellitus	J40-J47	Chronic diseases of the lower respiratory tract	V00	Traffic accidents and transportation
	I26-I28	Cardiopulmonary diseases and pulmonary circulation diseases	J95-J99	Other respiratory system diseases	W00-W99, X00-X59	Other external causes of accidents (unintentional)
	I30-I52	Forms of heart diseases	N17-N19	Kidney failure		
	J09-J18	Influenza and pneumonia	R95-R99	Death due to unknown and unknown reasons		
**Class 3**	C00-C75	Location except lymph, hematopoietic system and related tissue	I10-I15	High blood pressure disease	I20-I25	Ischemic heart diseases
	I60-I69	Vascular diseases of the brain				

**Fig. 5:**
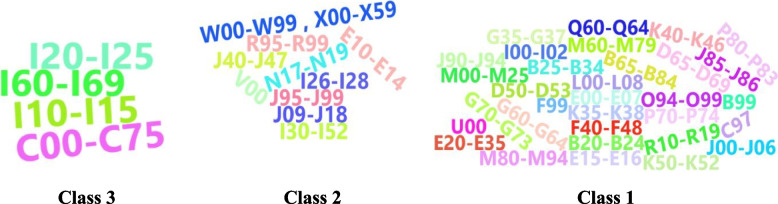
Representation of membership to classes 1 to 3 using LGMM

In addition, a word cloud plot (Fig. 5) was used to visually represent the members of each class. The parameters of the three-class linear LGMM were estimated and are presented in Table 3. The estimated mean intercept was found to be significant in all three classes, indicating a significant difference in the initial frequencies of causes of death among the classes. The estimated mean slope (108.07) was only significant in class 3, suggesting a substantial increase in the frequency of death caused within this class. Conversely, the variation in the frequency of death causes remained constant in the other two classes. These findings indicate a rising trend in these causes from 2015 to 2019, with potential implications for the future. Consequently, further research is warranted to explore these causes, associated factors, and implement more precise measures for control. Supplementary analyses were conducted for different sex and age groups, and the results are presented in Tables A2, A3, A4 and A5 and Figures A1 and A2 in the supplementary material section. Table A2 reveals that the optimal number of classes was determined to be two.

**Table 3:** Estimation of LGMM’s parameters

Model	Class	Parameters	Estimate (S. E)	*P*-value
Unconditional	1	Intercept	68.79 (23.40)	0.003*
		Slope	1.30 (7.04)	0.853
	2	Intercept	874.18 (74.28)	< 0.001**
		Slope	14.18 (22.06)	0.520
	3	Intercept	2752.57 (115.30)	< 0.001**
		Slope	108.07 (34.76)	0.001

^**^ Significant in 0.001 error level; * significant in 0.05 error level

According to Table A3, the significant estimated mean intercept in two classes for both female and male groups indicates differences in the initial frequencies of causes of death among the classes. Additionally, the estimated mean slope (55.92) was found to be significant only in class two for the male group, suggesting a substantial increase in the frequency of death caused within this class. These findings suggest that mortality rates are higher in males compared to females. The analysis of age groups revealed that causes of death could be categorized into three classes across all age groups (Table A4). Table A5 further demonstrated significant differences in the initial frequencies of causes of death among the three classes for all age groups, starting from 2015. In the age group of less than one year, both classes 1 and 3 exhibited significant mean slope estimates, indicating a decreasing trend in the frequencies of causes of death from 2014 to 2018. In contrast, class 2 showed constant changes in frequencies. For the age groups of 2 to 14 and more than 65 years, the mean slope estimates were non-significant, indicating constant changes in these age groups. In the age groups of 15 to 24 and 25 to 44 years, class 1 showed a decreasing pattern in changes in frequencies of causes of death, while the other two classes exhibited constant changes.

## Discussion

One of the sustainable development goals is good health and well-being [23]. A decrease in mortality is considered a sign of a healthy community. The study of death and its causes is an essential topic worldwide, and this research can help in seeking a more sustainable future for the population's health. As a consequence, it can improve social welfare, life satisfaction, and quality of life [23]. Health promotion includes health education, health protection, and disease prevention. Achieving health promotion requires planning, increasing people's knowledge and skills, creating policies that support health, and more education by government and non-governmental institutions to improve people's lives by 2030 [23, 24]. Therefore, without the implementation of these policies and more research, there is no hope of improving people's health. High-quality research and precise results require collecting high-quality data in a timely manner.

To distinguish the underlying trends of the causes of individuals' death and longitudinal changes in heterogeneous subgroups, we analyzed data from 2015 to 2019 extracted from Iran's most comprehensive system for registering causes of death, which is a strength of this study. We clustered 112 causes of death into three classes. The pattern of changes in mortality due to diseases was constant (87.50%). Second-class diseases had a slightly upward trend (8.92%), and third-class diseases had a completely upward trend (3.57%).

Borumandnia et al. clustered 63 death causes among Iranian men from 1990 to 2016 into four classes. They found that the defined trend in mortality rates over time was increasing, slowly decreasing, stable slowly increasing, and almost sharp trend in classes 1 to 4, respectively. They also found that the non-linear growth mixture model was not significant [25].

According to the current study's findings, except for lymph, hematopoietic system and related tissue, vascular diseases of the brain, high blood pressure disease, and ischemic heart diseases, all other locations showed a completely upward trend (slop = 108.07 and *P*-value < 0.001) over time.

Ischemic heart disease had a slowly decreasing trend from 1990 to 2016 in Borumandnia et al. study, while after that (from 2015 to 2019), it showed a completely upward trend. High blood pressure is a risk factor and a major cause of CVD and mortality [26].

In 2019, high systolic blood pressure was reported as a leading cause of death, accounting for nearly 10.8 million deaths worldwide [27]. Additionally, hypertension affected 1.28 billion individuals in 2019 [28].

Lifestyle modifications have been shown to effectively improve cardiovascular disease risk factors, as demonstrated in numerous studies and reviews. However, it is important to note that advanced age and higher body mass index are associated with an increased risk of hypertension [29].

Our study revealed that several factors, including diabetes mellitus, cardiopulmonary diseases, pulmonary circulation diseases, various forms of heart disease, influenza and pneumonia, chronic lower respiratory tract diseases, other respiratory system diseases, kidney failure, death due to unknown causes, traffic accidents and transportation-related incidents, and other unintentional external causes of accidents, were classified as second-class factors. These factors exhibited a slight upward trend with a slope of 14.18, but the *P*-value was not significant (*P* = 0.520).

Patients with type II diabetes and metabolic syndrome are at an increased risk of developing cardiovascular disease and related events, thereby reducing life expectancy. Consumption of a high-salt, hypercaloric-high-carbohydrate diet leads to hypertension and hyperinsulinemia, resulting in obesity that further aggravates cardiovascular disease. Therefore, dietary interventions are essential to prevent these outcomes and should not be ignored [29]. Studies have shown that long-term weight loss, adoption of a low-calorie dietary pattern, reduction in blood pressure, and use of anti-hypertensive drugs can be beneficial for patients with type 2 diabetes [29].

Boroujeni et al. utilized latent growth mixture modeling (LGMM) to identify different longitudinal trends in lung cancer incidence in Europe from 1990 to 2016. They performed LGMM on male and female sub-groups separately and found that the overall pattern of incidence related to female and male lung cancer was rising and falling, respectively [30].

A systematic analysis of the Global Burden of Disease (GBD) from 1990 to 2015 highlighted the importance of neurological disorders, which accounted for 6.3% of global Disability-Adjusted Life-Years (DALYs). Neurological disorders caused 9.399 million deaths in 2015, accounting for 16.8% of global deaths [31].

Ghadirzadeh et al. showed that between 2001 and 2010, an annual average of 34.6 per hundred thousand people were killed in traffic accidents, with more than 80% of the casualties being men, and a descending trend over time [32]. However, recent data suggest that traffic accidents have experienced a slightly upward trend, indicating the need for increased attention.

Kidney disease is one of the most common chronic diseases with a global prevalence above 10% and a slight upward trend in the second class. It is associated with other chronic diseases, such as obesity, diabetes, and hypertension. Modifying lifestyle, controlling blood pressure, and using anti-hypertensive medication is recommended to reduce the risk of renal failure [33].

According to WHO’s reports, Air pollutants are responsible for 4.2 million premature deaths and various diseases, including 29% of lung cancer, 25% of ischemic heart disease, 17% of acute lower respiratory infections, 24% of stroke, and 43% of chronic obstructive pulmonary disease [34]. The recent outbreak of COVID-19, a respiratory infection disease, significantly increased the number of deaths [8].

With regards to the discussions surrounding the significance of serious and chronic diseases that result in mortality, as well as the identified increasing trend, there is a pressing need for more rigorous measures to be taken. The primary recommendation is the modification of lifestyle, which plays a prominent role in preventing and controlling diseases. The World Health Organization (WHO) has projected that lifestyle-related diseases are the cause of 70% to 80% of mortalities in developed countries and 40% to 50% in developing countries [35].

In their systematic review study conducted in Iran, Ghanaei et al. found that a poor lifestyle is a significant factor, accounting for 53% of deaths, in the incidence of chronic diseases such as colon cancer, hypertension, chronic obstructive pulmonary diseases, hepatic cirrhosis, HIV, and CVD. Adopting a healthier lifestyle can overcome many major risk factors, promote health, and reduce mortality [35]. In addition to a poor lifestyle, the structural weaknesses of health policy-makers, inadequate attention to general health education, and insufficient educational content on health promotion are important and critical issues that require basic planning to be implemented.

Gender and age are significant factors that impact the prevalence, burden of disease, and mortality rates globally. Neurological disorders exhibit a 10% difference in death and DALY rates between males and females, with higher rates observed in males. The majority of the burden due to neurological disorders is borne by individuals in the age group of 0–5 years. Epilepsy is a disease that affects children and young adults and causes the most burden. Headaches peak between the ages of 25 to 49 years, while the burden of other neurological disorders increases with age. Stroke is the primary contributor to DALY. Geographical regions and their variations are also crucial as communicable neurological disorders are more prevalent in high-income regions and central Europe [31].

Other factors such as seasons and residential areas can also affect disease prevalence and mortality rates. Consequently, these factors may impact the clustering of death causes and their longitudinal trends over time. To obtain more accurate results and implement necessary policies in the field of public health to reduce mortality, larger scale studies are recommended, considering a larger sample size, other medical centers, regions, and provinces. Additionally, data analysis with bivariate or multivariate growth mixture models is recommended.

### Limitations

Several limitations must be considered in this study. Firstly, the small number of disease causes limited the use of mixed growth models. Secondly, the heterogeneity of diseases resulted in some classes having a small volume. Accurate and high-quality mortality data are crucial for informing public health policy. In some cases, the cause of death may remain unspecified or the cause of death may be recorded for another reason, limiting the discussion about the cause of death.

## Conclusion

Based on the current findings, identifying the rising trends of diseases leading to death using LGMM can be a suitable tool for the prevention and management of diseases. Chronic diseases such as high blood pressure, diabetes mellitus, chronic diseases of the lower respiratory tract, kidney failure, cardiopulmonary diseases, pulmonary circulation diseases, ischemic heart diseases, traffic accidents, and transportation have been increasing up to 2019, which can serve as a warning for health policymakers in society.

## Supplementary Information

**Additional file 1: Table A1.** Number of deaths by cause in each year. **Table A2.** Fitted indices for LGMMs by sex with 1 to 3 classes. **Table A3. **Estimation of LGMM’s parameters by sex. **Table A4.** Fitted indices for LGMMs by age with 1 to 3 classes. **Table A5. **Estimation of LGMM’s parameters by age.

**Additional file 2: Figure A1.** Representation of membership to classes 1 and 2 using LGMM by sex. **Figure A2.** Representation of membership to classes 1 to 3 using LGMM by age.

## Data Availability

The datasets used and analyzed during the current study are available from the corresponding author on reasonable request.

## Abbreviations

LGMM: Latent Growth Mixture Models
ICD-10: International Classification of Diseases 10th Revision
WHO: World Health Organization
AIC: Akaike Information Criterion
BIC: Bayesian Information Criterion
LRT: Likelihood Ratio Test
GBD: Global Burden of Disease
DALY: Disability-Adjusted Life-Years
