# Supplementary material for: Clustering of the causes of death in Northeast Iran: a mixed growth modeling
Source: BMC Public Health. 2023 Jul 19;23:1384. doi: 10.1186/s12889-023-16245-y (PMC10355030; doi:10.1186/s12889-023-16245-y)
Supplement: Supplementary file 1 — Additional file 1: Table A1. Number of deaths by cause in each year. Table A2. Fitted indices for LGMMs by sex with 1 to 3 classes. Table A3. Estimation of LGMM’s parameters by sex. Table A4. Fitted indices for LGMMs by age with 1 to 3 classes. Table A5. Estimation of LGMM’s parameters by age. [file 12889_2023_16245_MOESM1_ESM.docx]

--------------------------------------------------- **Appendix** ----------------------------------------------------

**Table A1.** Number of deaths by cause in each year

| ICD-10 code | Year | | | | | Total |
| --- | --- | --- | --- | --- | --- | --- |
|  | **2015** | **2016** | **2017** | **2018** | **2019** |  |
| E00-E07 | 8 | 2 | 6 | 7 | 7 | 30 |
| L00-L08 | 3 | 7 | 8 | 11 | 5 | 34 |
| K35-K38 | 9 | 9 | 6 | 7 | 4 | 35 |
| B65-B84 | 23 | 2 | 5 | 3 | 7 | 40 |
| F40-F48 | 8 | 2 | 11 | 9 | 11 | 41 |
| B25-B34 | 6 | 11 | 13 | 4 | 8 | 42 |
| B20-B24 | 9 | 14 | 6 | 6 | 9 | 44 |
| M60-M79 | 10 | 9 | 12 | 9 | 5 | 45 |
| D50-D53 | 15 | 5 | 13 | 6 | 9 | 48 |
| O94-O99 | 12 | 12 | 7 | 8 | 9 | 48 |
| G60-G64 | 9 | 4 | 17 | 9 | 11 | 50 |
| F99 | 7 | 6 | 10 | 18 | 13 | 54 |
| P70-P74 | 10 | 13 | 6 | 18 | 7 | 54 |
| E15-E16 | 13 | 15 | 9 | 8 | 12 | 57 |
| R10-R19 | 19 | 15 | 11 | 6 | 7 | 58 |
| D65-D69 | 14 | 11 | 16 | 14 | 6 | 61 |
| Q60-Q64 | 17 | 9 | 13 | 16 | 8 | 63 |
| I00-I02 | 24 | 5 | 2 | 25 | 11 | 67 |
| M80-M94 | 15 | 13 | 9 | 14 | 16 | 67 |
| K40-K46 | 11 | 17 | 14 | 16 | 10 | 68 |
| G70-G73 | 9 | 15 | 10 | 17 | 18 | 69 |
| G35-G37 | 11 | 6 | 19 | 22 | 12 | 70 |
| K50-K52 | 18 | 6 | 15 | 17 | 16 | 72 |
| M00-M25 | 8 | 12 | 15 | 17 | 20 | 72 |
| D70-D77 | 13 | 24 | 9 | 8 | 23 | 77 |
| N40-N51 | 14 | 14 | 21 | 19 | 10 | 78 |
| N10-N16 | 18 | 12 | 12 | 20 | 19 | 81 |
| J85-J86 | 23 | 34 | 13 | 6 | 8 | 84 |
| P75-P78 | 20 | 16 | 11 | 22 | 16 | 85 |
| G10-G13 | 17 | 19 | 21 | 14 | 19 | 90 |
| Q30-Q34 | 32 | 22 | 21 | 22 | 15 | 112 |
| E20-E35 | 50 | 19 | 22 | 11 | 11 | 113 |
| J90-J94 | 23 | 32 | 10 | 29 | 20 | 114 |
| P80-P83 | 24 | 15 | 25 | 25 | 25 | 114 |
| Q90-Q99 | 20 | 30 | 16 | 30 | 26 | 122 |
| Y35-Y36 | 46 | 38 | 21 | 6 | 11 | 122 |
| E50-E68 | 23 | 16 | 32 | 31 | 21 | 123 |
| R40-R46 | 20 | 49 | 20 | 24 | 11 | 124 |
| Q65-Q79 | 33 | 32 | 26 | 16 | 25 | 132 |
| J20-J22 | 18 | 23 | 41 | 16 | 40 | 138 |
| B99 | 57 | 39 | 18 | 18 | 11 | 143 |
| D60-D64 | 32 | 41 | 32 | 18 | 23 | 146 |
| M30-M36 | 34 | 30 | 26 | 32 | 26 | 148 |
| J00-J06 | 23 | 18 | 36 | 25 | 47 | 149 |
| Y85-Y89 | 56 | 37 | 27 | 22 | 14 | 156 |
| N30-N39 | 17 | 29 | 25 | 50 | 48 | 169 |
| Q38-Q45 | 51 | 30 | 27 | 31 | 31 | 170 |
| P35-P39 | 29 | 44 | 29 | 46 | 30 | 178 |
| P50-P61 | 51 | 43 | 38 | 32 | 19 | 183 |
| G20-G26 | 18 | 35 | 50 | 39 | 44 | 186 |
| G00-G09 | 34 | 33 | 45 | 37 | 39 | 188 |
| E40-E46 | 6 | 56 | 37 | 84 | 10 | 193 |
| 180-I89 | 42 | 38 | 39 | 45 | 31 | 195 |
| F00-F09 | 62 | 38 | 29 | 35 | 45 | 209 |
| R00-R09 | 51 | 33 | 32 | 64 | 33 | 213 |
| D55-D59 | 39 | 51 | 28 | 38 | 60 | 216 |
| I05-I09 | 76 | 38 | 49 | 34 | 26 | 223 |
| C97 | 41 | 31 | 37 | 44 | 71 | 224 |
| I95-I99 | 80 | 46 | 49 | 30 | 33 | 238 |
| K65-K67 | 33 | 42 | 57 | 59 | 53 | 244 |
| G40-G47 | 62 | 52 | 43 | 44 | 52 | 253 |
| D00-D09 | 45 | 43 | 60 | 50 | 57 | 255 |
| N25-N29 | 64 | 57 | 59 | 49 | 41 | 270 |
| J30-J39 | 23 | 17 | 230 | 12 | 10 | 292 |
| U00 | 0 | 0 | 1 | 1 | 293 | 295 |
| B15-B19 | 48 | 58 | 92 | 59 | 50 | 307 |
| Q80-Q89 | 67 | 53 | 66 | 65 | 60 | 311 |
| Q00-Q07 | 95 | 63 | 51 | 61 | 60 | 330 |
| L80-L99 | 38 | 50 | 55 | 77 | 126 | 346 |
| Y40-Y84 | 134 | 56 | 56 | 64 | 36 | 346 |
| K20-K31 | 68 | 61 | 50 | 81 | 97 | 357 |
| N00-N08 | 105 | 123 | 92 | 54 | 29 | 403 |
| K80-K87 | 72 | 102 | 75 | 84 | 73 | 406 |
| J60-J70 | 71 | 62 | 89 | 95 | 118 | 435 |
| G80-G83 | 51 | 94 | 105 | 98 | 133 | 481 |
| J80-J84 | 114 | 112 | 123 | 80 | 57 | 486 |
| E70-E90 | 102 | 105 | 118 | 104 | 91 | 520 |
| A15-A19 | 110 | 112 | 91 | 111 | 110 | 534 |
| G90-G99 | 102 | 101 | 132 | 110 | 92 | 537 |
| D10-D36 | 91 | 99 | 124 | 106 | 122 | 542 |
| X85-X99 , Y00-Y09 | 114 | 83 | 97 | 130 | 152 | 576 |
| Y10-Y34 | 198 | 118 | 181 | 50 | 41 | 588 |
| A00-A09 | 110 | 219 | 142 | 52 | 66 | 589 |
| F10-F19 | 44 | 146 | 109 | 176 | 150 | 625 |
| K55-K63 | 106 | 142 | 114 | 141 | 123 | 626 |
| P00-P04 | 142 | 123 | 105 | 114 | 249 | 733 |
| G30-G32 | 159 | 178 | 191 | 148 | 194 | 870 |
| K90-K93 | 130 | 168 | 203 | 192 | 180 | 873 |
| X60-X84 | 212 | 220 | 220 | 244 | 220 | 1116 |
| Q20-Q28 | 268 | 242 | 280 | 222 | 226 | 1238 |
| I70-I79 | 185 | 165 | 171 | 150 | 703 | 1374 |
| C76-C80 | 214 | 230 | 349 | 292 | 293 | 1378 |
| A30-A49 | 222 | 215 | 366 | 390 | 245 | 1438 |
| P90-P96 | 313 | 215 | 221 | 203 | 489 | 1441 |
| K70-K77 | 220 | 284 | 327 | 280 | 339 | 1450 |
| P20-P29 | 354 | 371 | 409 | 316 | 350 | 1800 |
| R50-R69 | 390 | 412 | 505 | 391 | 245 | 1943 |
| P05-P08 | 562 | 561 | 473 | 380 | 446 | 2422 |
| N17-N19 | 365 | 529 | 627 | 661 | 575 | 2757 |
| R95-R99 | 568 | 731 | 679 | 531 | 482 | 2991 |
| I26-I28 | 1070 | 584 | 806 | 372 | 283 | 3115 |
| J95-J99 | 621 | 574 | 648 | 691 | 581 | 3115 |
| W00-W99 , X00-X59 | 969 | 707 | 731 | 819 | 806 | 4032 |
| J40-J47 | 655 | 675 | 898 | 969 | 1143 | 4340 |
| J09-J18 | 830 | 840 | 995 | 1181 | 1649 | 5495 |
| E10-E14 | 860 | 1165 | 1416 | 1219 | 1228 | 5888 |
| V00 | 1565 | 1366 | 1242 | 1301 | 1157 | 6631 |
| I30-I52 | 1380 | 1318 | 1863 | 1596 | 1284 | 7441 |
| I10-I15 | 2098 | 2239 | 1879 | 1918 | 2537 | 10671 |
| I60-I69 | 2260 | 2252 | 2622 | 2350 | 2440 | 11924 |
| C00-C75 | 3168 | 3414 | 3648 | 3592 | 3791 | 17613 |
| I20-I25 | 3821 | 4078 | 4281 | 4692 | 4456 | 21328 |

**Table A2.** Fitted indices for LGMMs by sex with 1 to 3 classes.

| Sex | Class | AIC | BIC | Entropy | P-value (LRT test) | Percent in class | | |
| --- | --- | --- | --- | --- | --- | --- | --- | --- |
|  |  |  |  |  |  | **Class1** | **Class2** | **Class3** |
| Female | 1 | 6641.99 | 6649.29 | 1.00 | - | 100% |  |  |
|  | 2 | 5980.91 | 5996.11 | 1.00 | <0.001** | 95.69% | 4.30% |  |
|  | 3 | 5685.38 | 5708.18 | 0.99 | <0.001** | 4.30% | 88.17% | 7.52% |
| Male | 1 | 74.20.48 | 7428.32 | 1.00 | - | 100% |  |  |
|  | 2 | 6804.31 | 6820.00 | 0.99 | <0.001** | 5.94% | 94.05% |  |
|  | 3 | 6341.37 | 6364.91 | 0.99 | <0.001** | 7.92% | 1.98% | 90.09% |

** Significant at 0.001 error level; * significant in 0.05 error level

**Table A3.** Estimation of LGMM’s parameters by sex

| Sex | Class | Parameters | Estimate (S. E) | P-value |
| --- | --- | --- | --- | --- |
| Female | 1 | Intercept | 68.76 (15.91) | <0.001** |
|  |  | Slope | 2.34 (4.79) | 0.625 |
|  | 2 | Intercept | 1226.92 (75.05) | <0.001** |
|  |  | Slope | 55.92 (22.63) | 0.013* |
| Male | 1 | Intercept | 1367.63 (82.49) | <0.001** |
|  |  | Slope | 21.23 (24.87) | 0.393 |
|  | 2 | Intercept | 74.92 (20.75) | <0.001** |
|  |  | Slope | 1.52 (6.25) | 0.807 |

** Significant in 0.001 error level; * significant in 0.05 error level.

**Table A4.** Fitted indices for LGMMs by age with 1 to 3 classes.

| Age | Class | AIC | BIC | Entropy | P-value (LRT test) | Percent in class | | |
| --- | --- | --- | --- | --- | --- | --- | --- | --- |
|  |  |  |  |  |  | **Class1** | **Class2** | **Class3** |
| Age < 1 | 1 | 1617.92 | 1622.41 | 1.00 | - | 100% |  |  |
|  | 2 | 1416.33 | 1425.31 | 1.00 | <0.001** | 87.87% | 12.12% |  |
|  | 3 | 1315.29 | 1328.76 | 0.99 | <0.001** | 6.06% | 87.87% | 6.06% |
| 2 < Age < 14 | 1 | 506.94 | 508.64 | 1.00 | - | 100% |  |  |
|  | 2 | 422.32 | 425.70 | 0.99 | <0.001** | 15.38% | 84.61% |  |
|  | 3 | 421.74 | 426.82 | 0.88 | <0.001** | 15.38% | 23.07% | 61.53% |
| 15 < Age < 24 | 1 | 612.92 | 614.84 | 1.00 | - | 100% |  |  |
|  | 2 | 532.95 | 536.78 | 1.00 | <0.001** | 92.95% | 7.14% |  |
|  | 3 | 480.23 | 485.98 | 0.99 | <0.001** | 7.14% | 78.57% | 14.28% |
| 25 < Age < 44 | 1 | 1842.48 | 1847.47 | 1.00 | - | 100% |  |  |
|  | 2 | 1641.90 | 1651.89 | 0.99 | <0.001** | 10.25% | 89.74% |  |
|  | 3 | 1561.83 | 1576.80 | 0.98 | <0.001** | 7.69% | 79.48% | 12.82% |
| 45 < Age < 64 | 1 | 2559.89 | 2565.44 | 1.00 | - | 100% |  |  |
|  | 2 | 2316.44 | 2327.55 | 1.00 | <0.001** | 4.25% | 95.4% |  |
|  | 3 | 2094.53 | 2111.18 | 0.99 | <0.001** | 85.10% | 10.63% | 4.25% |
| Age > 65 | 1 | 4320.27 | 4327.02 | 1.00 | - | 100% |  |  |
|  | 2 | 3918.83 | 3932.32 | 1.00 | <0.001** | 94.28% | 5.71% |  |
|  | 3 | 3764.33 | 3784.57 | 0.99 | <0.001** | 87.14% | 5.71% | 7.14% |

** Significant at 0.001 error level; * significant in 0.05 error level

**Table A5.** Estimation of LGMM’s parameters by age

| Age(year) | Class | Parameters | Estimate (S. E) | P-value |
| --- | --- | --- | --- | --- |
| Age < 1 | 1 | Intercept | 514.74 (25.47) | <0.001** |
|  |  | Slope | -34.94 (9.30) | <0.001** |
|  | 2 | Intercept | 35.32 (6.69) | <0.001** |
|  |  | Slope | -2.73 (2.44) | 0.263 |
|  | 3 | Intercept | 281.24 (25.48) | <0.001** |
|  |  | Slope | -19.69 (9.30) | 0.034* |
| 2 < Age < 14 | 1 | Intercept | 99.50 (8.29) | <0.001** |
|  |  | Slope | -1.70 (3.02) | 0.574 |
|  | 2 | Intercept | 25.15 (6.65) | <0.001** |
|  |  | Slope | 1.27 (2.66) | 0.631 |
|  | 3 | Intercept | 15.30 (4.50) | <0.001** |
|  |  | Slope | -0.35 (1.65) | 0.817 |
| 15 < Age < 24 | 1 | Intercept | 258.50 (15.59) | <0.001** |
|  |  | Slope | -16.10 (5.69) | 0.004* |
|  | 2 | Intercept | 20.63 (4.70) | <0.001** |
|  |  | Slope | -0.53 (1.71) | 0.754 |
|  | 3 | Intercept | 77.75 (11.03) | <0.001** |
|  |  | Slope | -0.29 (4.02) | 0.940 |
| 25 < Age < 44 | 1 | Intercept | 357.83 (20.62) | <0.001** |
|  |  | Slope | -15.93 (7.53) | 0.034* |
|  | 2 | Intercept | 18.79 (6.45) | 0.003* |
|  |  | Slope | 0.88 (2.33) | 0.703 |
|  | 3 | Intercept | 122.13 (16.68) | <0.001** |
|  |  | Slope | -2.25 (5.92) | 0.704 |
| 45 < Age < 64 | 1 | Intercept | 41.98 (10.32) | <0.001** |
|  |  | Slope | 0.87 (3.76) | 0.816 |
|  | 2 | Intercept | 314.29 (29.20) | <0.001** |
|  |  | Slope | 11.41 (10.66) | 0.284 |
|  | 3 | Intercept | 868.00 (46.17) | <0.001** |
|  |  | Slope | 54.69 (16.86) | 0.001** |
| Age > 65 | 1 | Intercept | 60.96 (27.27) | 0.025* |
|  |  | Slope | 3.06 (9.92) | 0.757 |
|  | 2 | Intercept | 1987.75 (105.95) | <0.001** |
|  |  | Slope | 74.80 (38.69) | 0.053 |
|  | 3 | Intercept | 607.00 (96.40) | <0.001** |
|  |  | Slope | 51.10 (34.27) | 0.135 |

** Significant in 0.001 error level; * significant in 0.05 error level.
