# Supplementary material for: Clustering of the causes of death in Northeast Iran: a mixed growth modeling
Source: BMC Public Health. 2023 Jul 19;23:1384. doi: 10.1186/s12889-023-16245-y (PMC10355030; doi:10.1186/s12889-023-16245-y)
Supplement: Supplementary file 2 — Additional file 2: Figure A1. Representation of membership to classes 1 and 2 using LGMM by sex. Figure A2. Representation of membership to classes 1 to 3 using LGMM by age. [file 12889_2023_16245_MOESM2_ESM.docx]

| 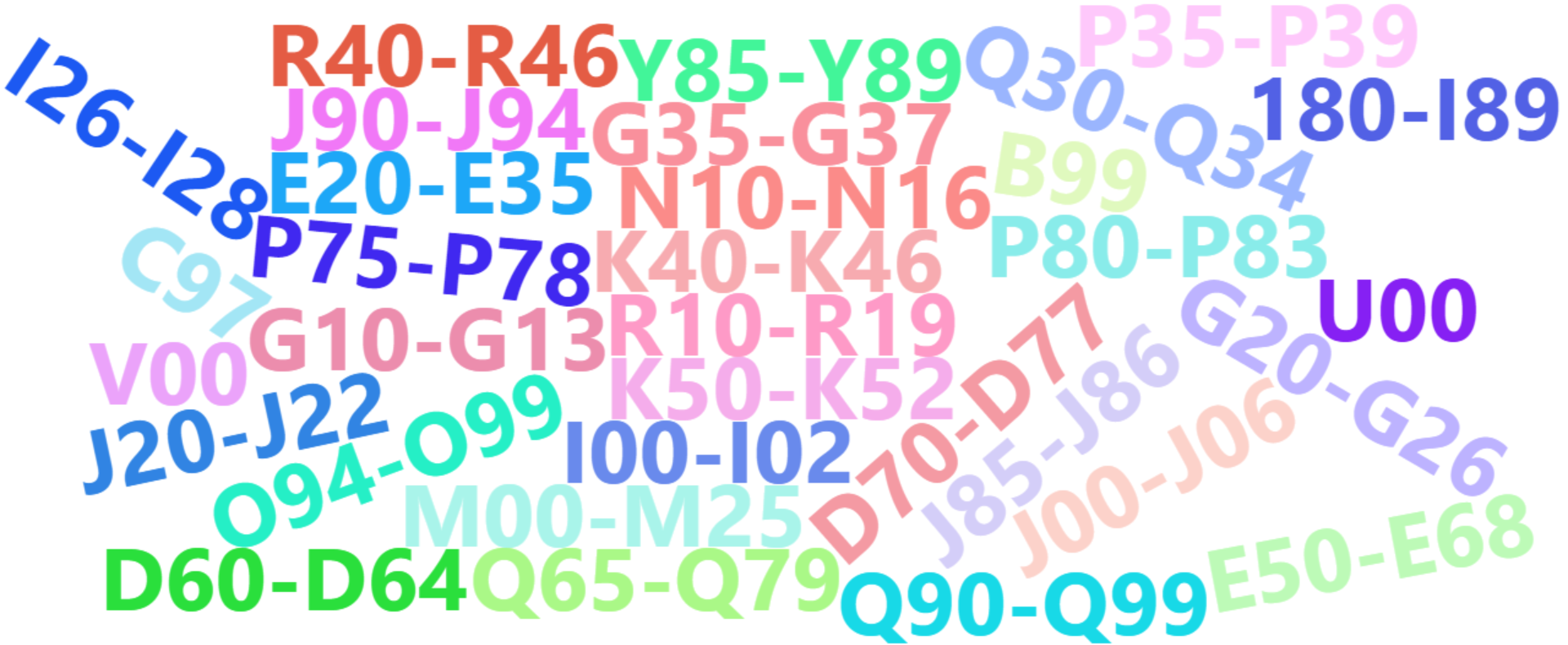 | | 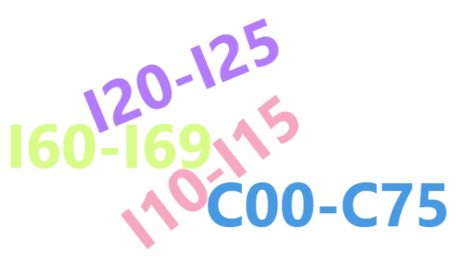 |
| --- | --- | --- |
| Class 1 | | **Class 2** |
| (A) Females | | |
| 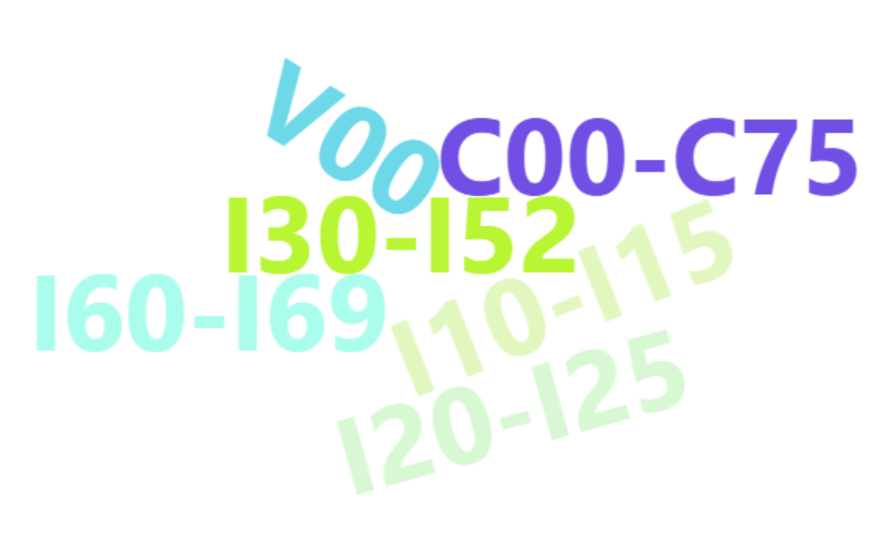 | 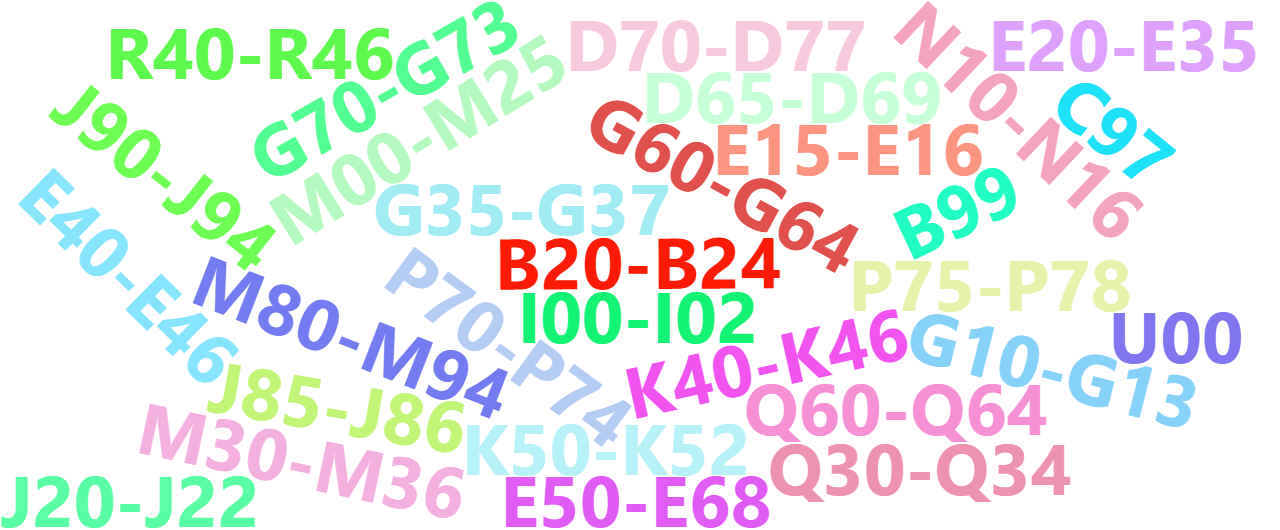 | |
| Class 1 | **Class 2** | |
| (B) Males | | |
| Figure A1. Representation of membership to classes 1 and 2 using LGMM by sex | | |

| 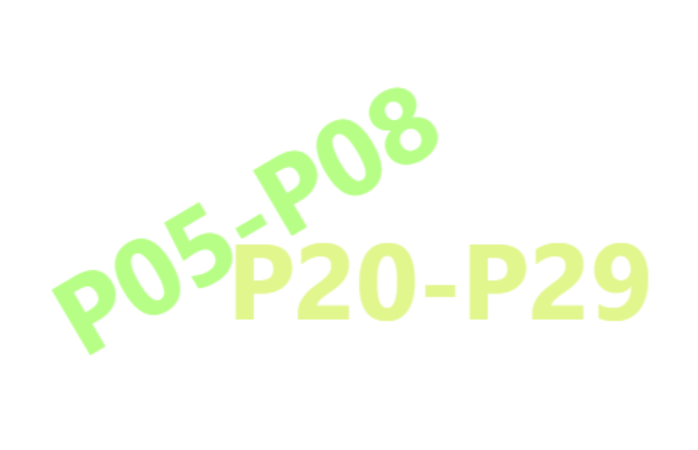 | | 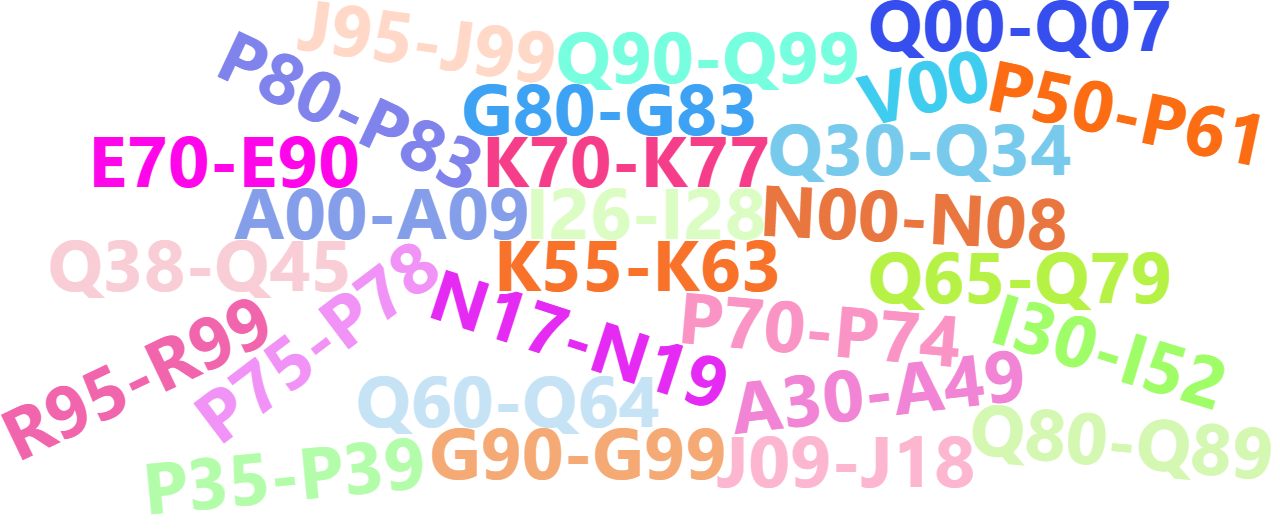 | | | | | | | 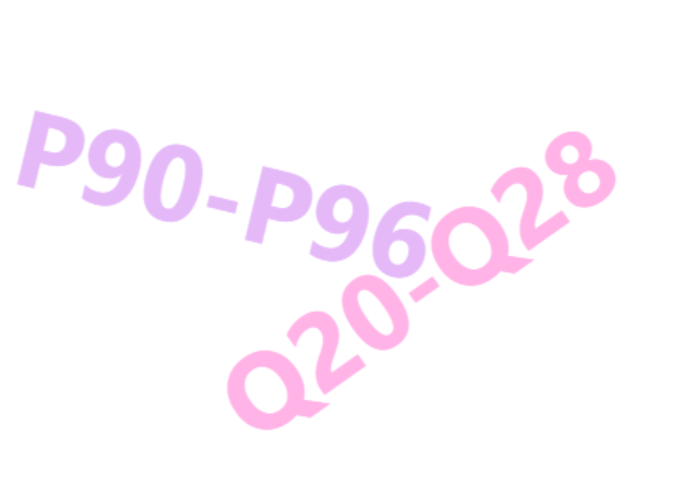 | | | |
| --- | --- | --- | --- | --- | --- | --- | --- | --- | --- | --- | --- | --- |
| Class 1 | | **Class 2** | | | | | | | **Class 3** | | | |
| (A) Age under 1 year | | | | | | | | | | | | |
| 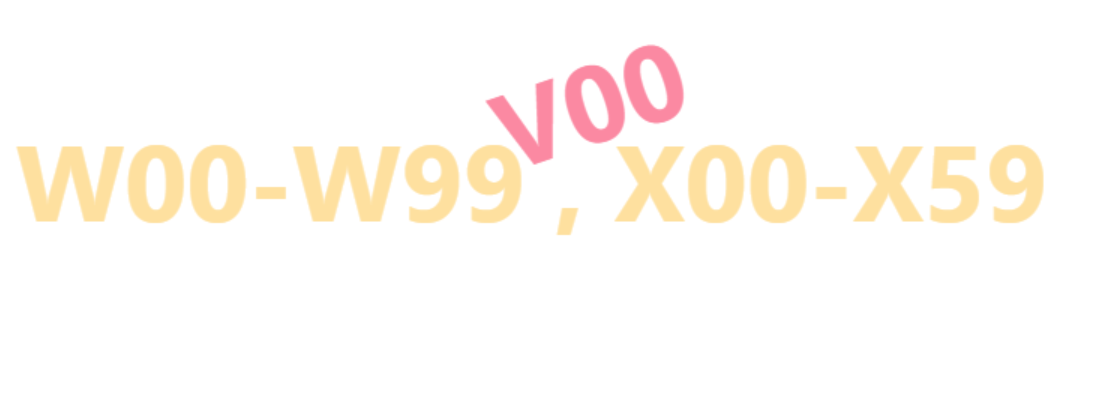 | | | 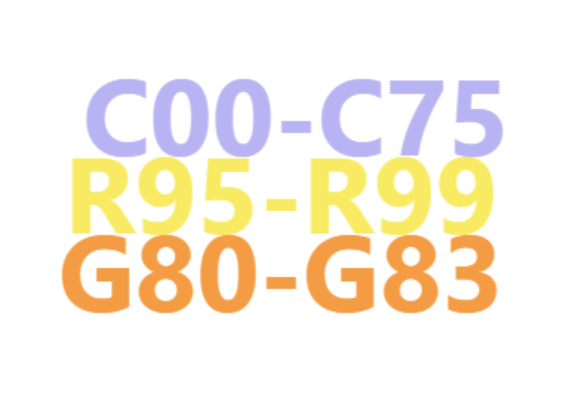 | | | | | 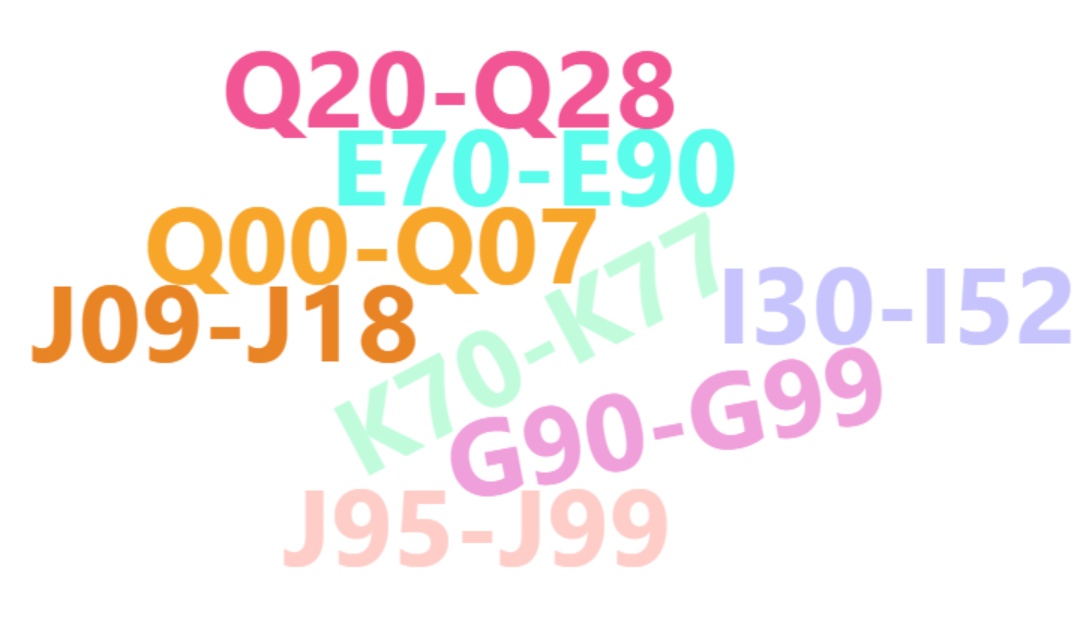 | | | | |
| Class 1 | | | **Class 2** | | | | | **Class 3** | | | | |
| (B) Age between 2 and 14 years | | | | | | | | | | | | |
| 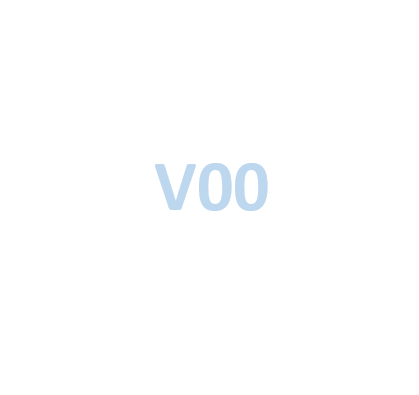 | 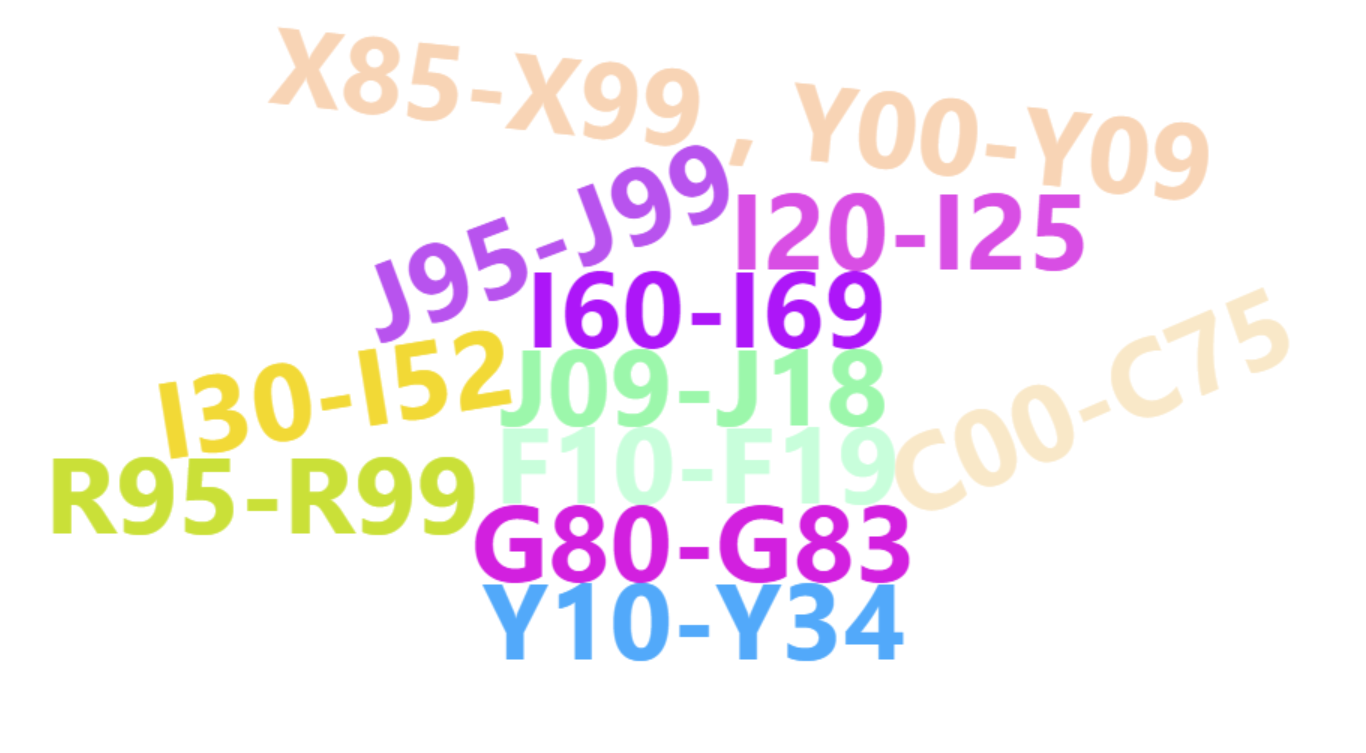 | | | | | | 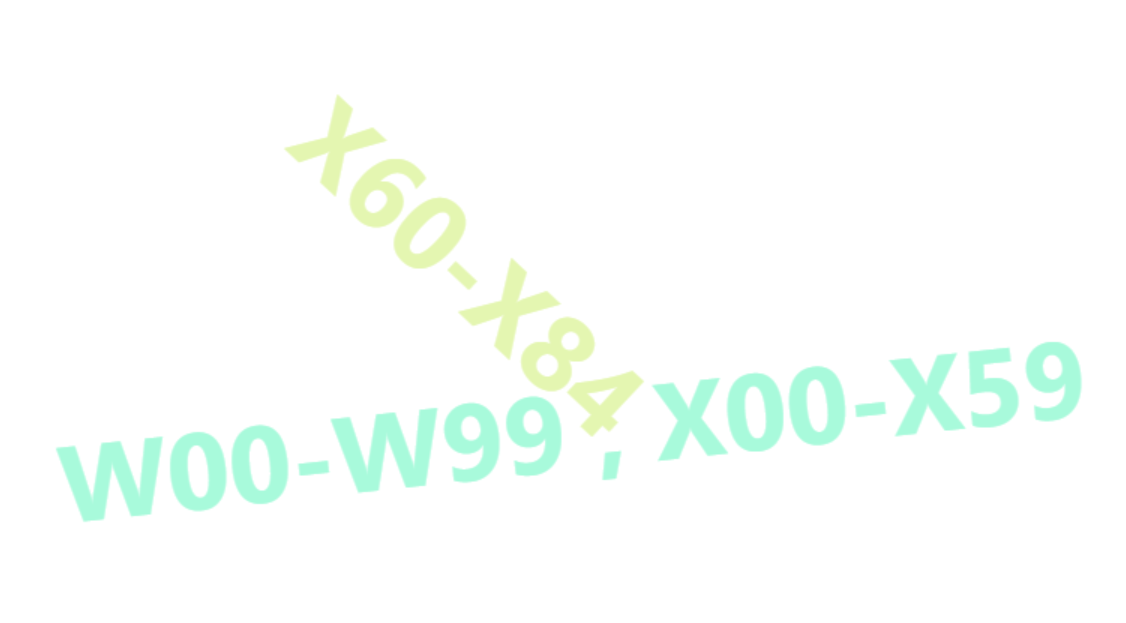 | | | | | |
| Class 1 | **Class 2** | | | | | | **Class 3** | | | | | |
| (C) Age between 15 and 24 years | | | | | | | | | | | | |
| 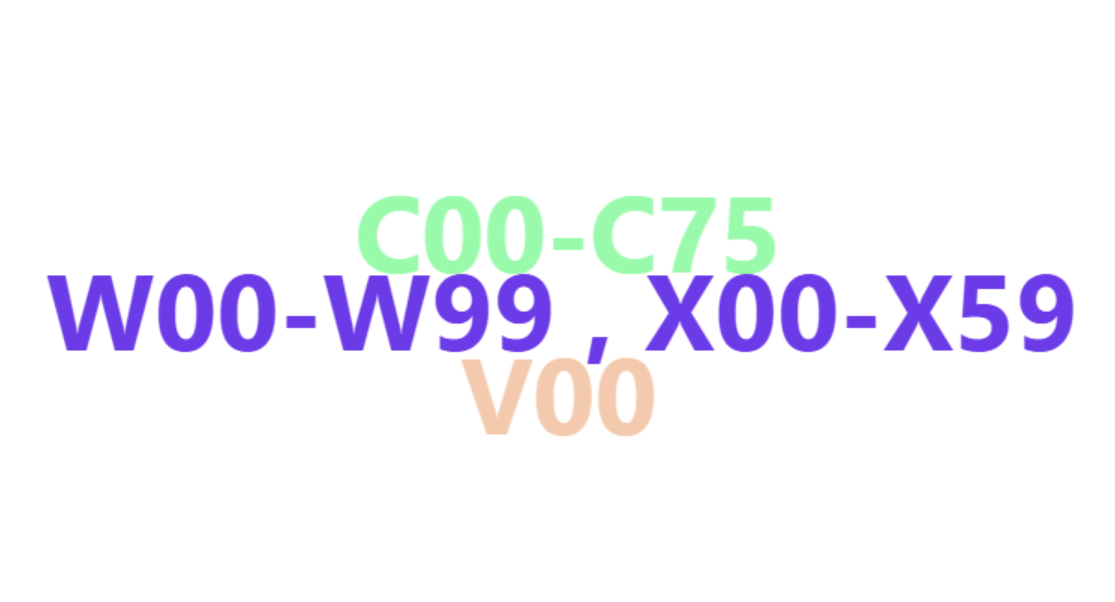 | | | | **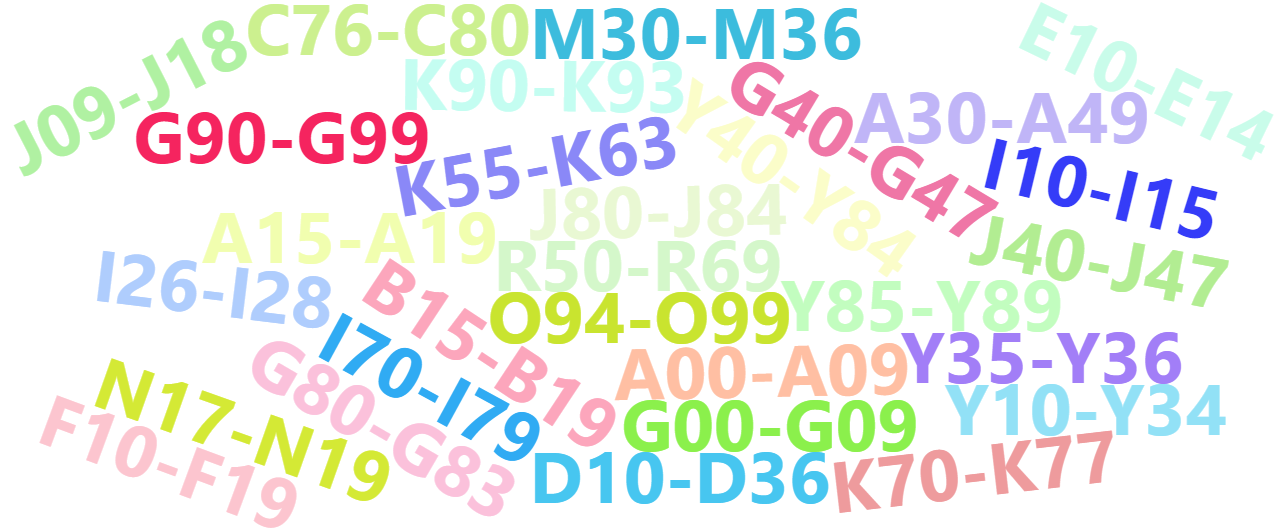** | | | | | | **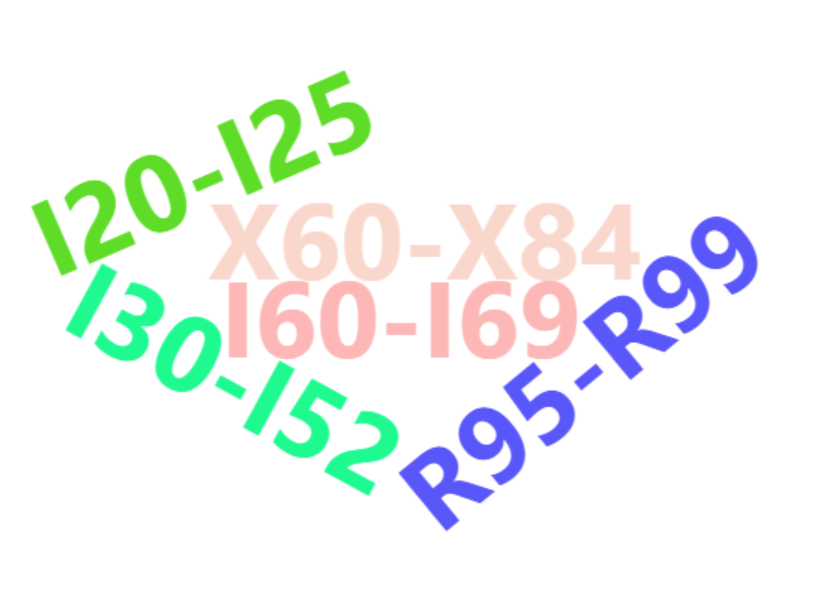** | | |
| Class 1 | | | | **Class 2** | | | | | | **Class 3** | | |
| (D) Age between 25 and 44 years | | | | | | | | | | | | |
| 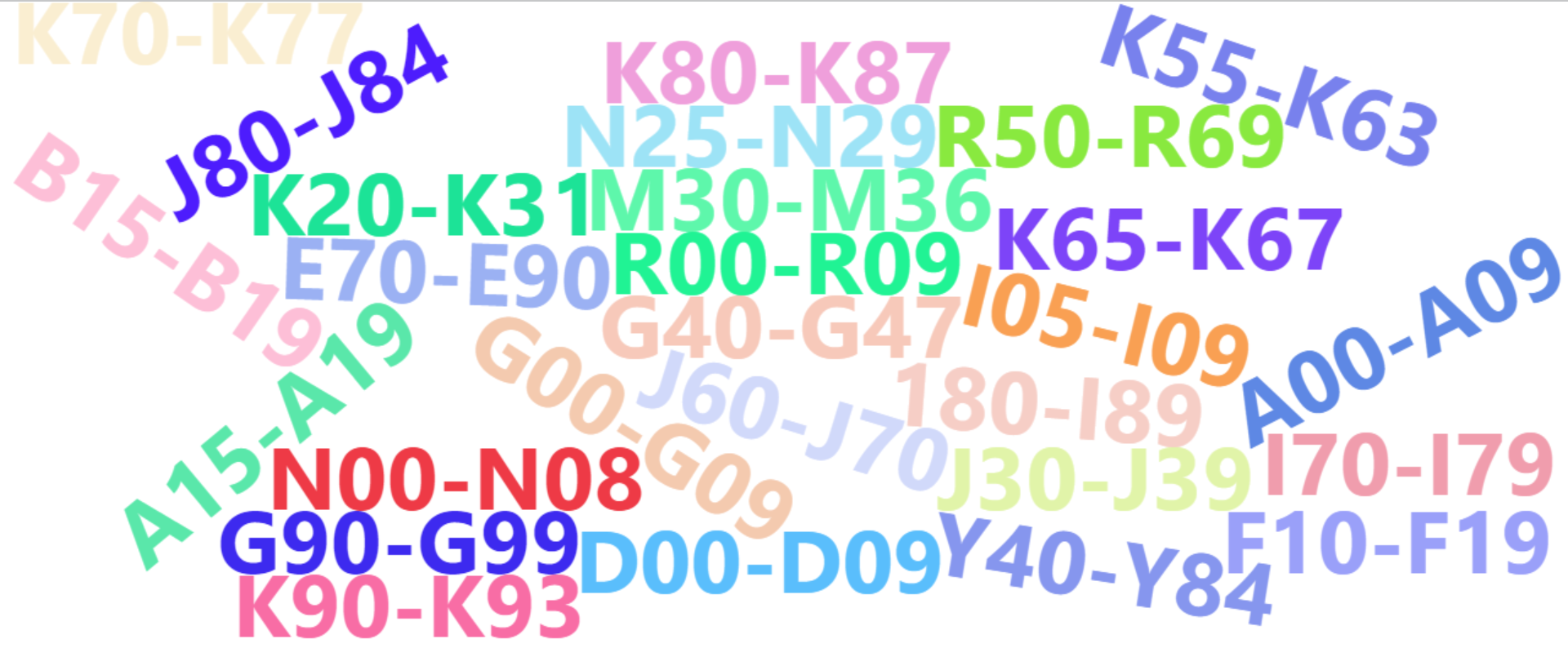 | | | | | **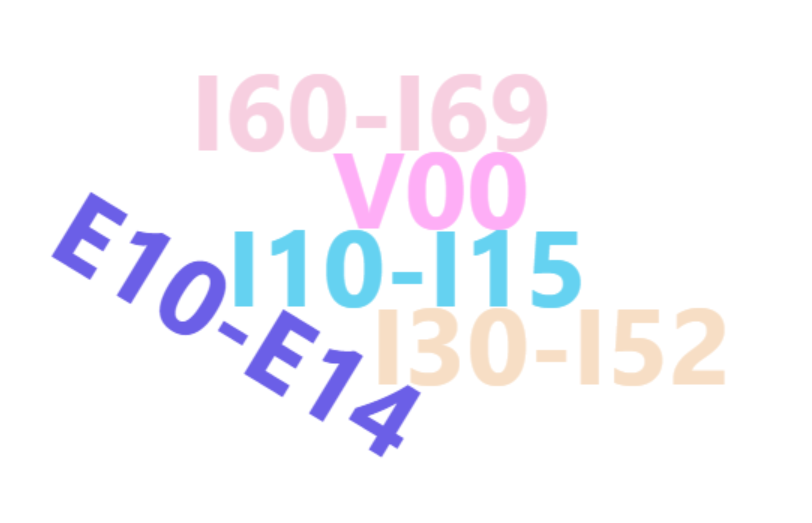** | | | | | | **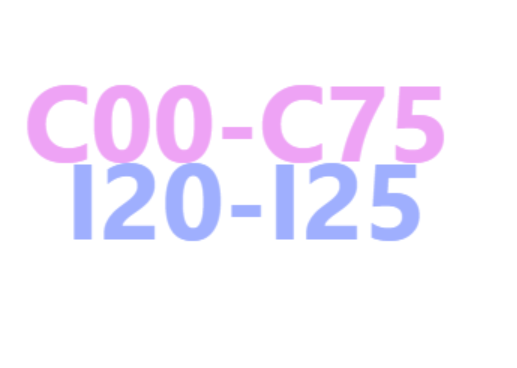** | |
| Class 1 | | | | | **Class 2** | | | | | | **Class 3** | |
| (E) Age between 45 and 64 years | | | | | | | | | | | | |
| 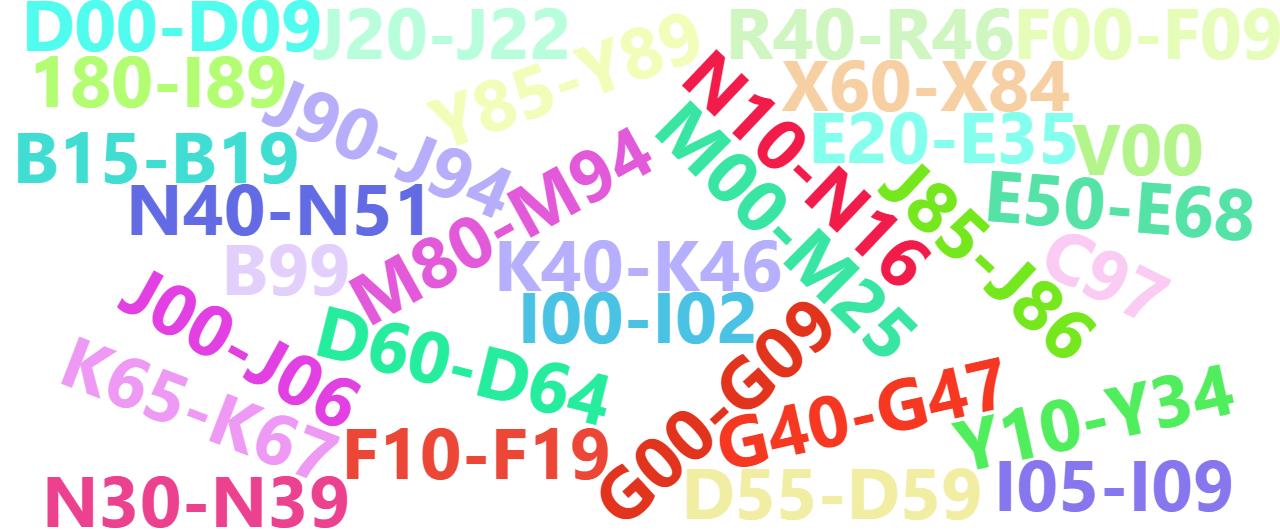 | | | | | | 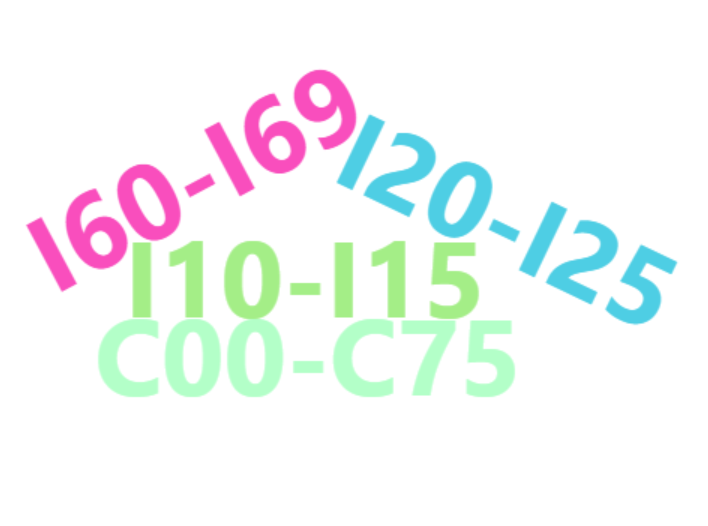 | | | | | | 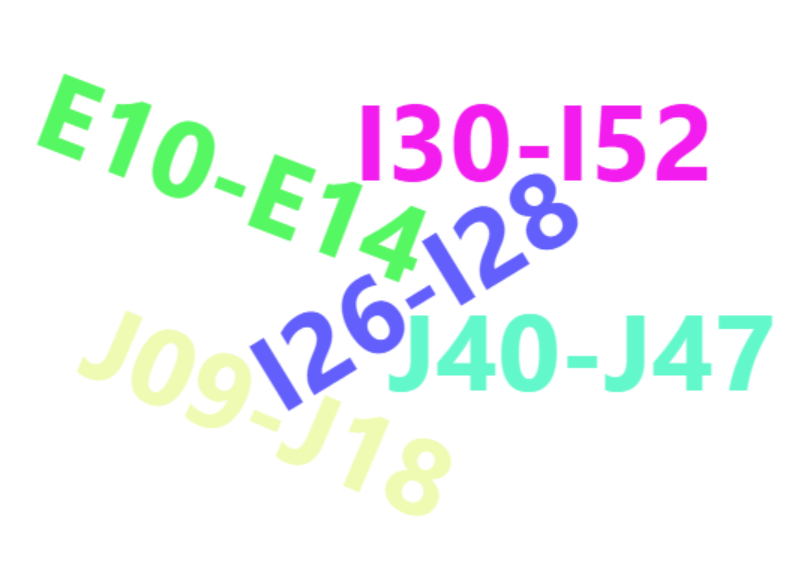 |
| Class 1 | | | | | | **Class 2** | | | | | | **Class 3** |
| (F) Age over 65 years | | | | | | | | | | | | |
| Figure A2. Representation of membership to classes 1 to 3 using LGMM by age | | | | | | | | | | | | |
